# Vitamin C—An Adjunctive Therapy for Respiratory Infection, Sepsis and COVID-19

**DOI:** 10.3390/nu12123760

**Published:** 2020-12-07

**Authors:** Patrick Holford, Anitra C. Carr, Thomas H. Jovic, Stephen R. Ali, Iain S. Whitaker, Paul E. Marik, A. David Smith

**Affiliations:** 1 Institute for Optimum Nutrition, Ambassador House, Richmond TW9 1SQ, UK; 2 Nutrition in Medicine Research Group, Department of Pathology & Biomedical Science, University of Otago, Christchurch 8140, New Zealand; anitra.carr@otago.ac.nz; 3 Reconstructive Surgery & Regenerative Medicine Research Group, Institute of Life Sciences, Swansea University Medical School, Swansea University, Swansea SA2 8PY, UK; Thomas.Jovic@wales.nhs.uk (T.H.J.); Stephen.Ali@wales.nhs.uk (S.R.A.); Iain.Whitaker@wales.nhs.uk (I.S.W.); 4 Welsh Centre for Burns & Plastic Surgery, Morriston Hospital, Swansea SA6 6NL, UK; 5 Division of Pulmonary and Critical Care Medicine, Eastern Virginia Medical School, Norfolk, VA 23507, USA; marikpe@evms.edu; 6 Department of Pharmacology, University of Oxford, Oxford OX1 3QT, UK; david.smith@pharm.ox.ac.uk

**Keywords:** COVID-19, SARS-CoV-2, coronavirus, vitamin C, ascorbate, colds, pneumonia, sepsis, immunonutrition, supplementation

## Abstract

There are limited proven therapies for COVID-19. Vitamin C’s antioxidant, anti-inflammatory and immunomodulating effects make it a potential therapeutic candidate, both for the prevention and amelioration of COVID-19 infection, and as an adjunctive therapy in the critical care of COVID-19. This literature review focuses on vitamin C deficiency in respiratory infections, including COVID-19, and the mechanisms of action in infectious disease, including support of the stress response, its role in preventing and treating colds and pneumonia, and its role in treating sepsis and COVID-19. The evidence to date indicates that oral vitamin C (2–8 g/day) may reduce the incidence and duration of respiratory infections and intravenous vitamin C (6–24 g/day) has been shown to reduce mortality, intensive care unit (ICU) and hospital stays, and time on mechanical ventilation for severe respiratory infections. Further trials are urgently warranted. Given the favourable safety profile and low cost of vitamin C, and the frequency of vitamin C deficiency in respiratory infections, it may be worthwhile testing patients’ vitamin C status and treating them accordingly with intravenous administration within ICUs and oral administration in hospitalised persons with COVID-19.

## 1. Introduction

Vitamin C, ascorbic acid, is an essential water-soluble nutrient. It is synthesised in plants from fructose and in almost all animals from glucose. It is not synthesised by primates, most bats, guinea pigs, and a small number of birds and fish since the final enzyme, gulonolactone oxidase (GULO), required for ascorbic acid synthesis is missing due to gene mutations that occurred prior to the evolution of Homo sapiens [1]. All these species are therefore dependent on vitamin C in their food. Primates are dependent on an adequate supply provided by fruits and vegetation intake ranging from 4.5 g/day for gorillas [2] to 600 mg/day for smaller monkeys (7.5 kg—a tenth of human size) [3].

The EU Average Requirement of 90 mg/day for men and 80 mg/day for women is to maintain a normal plasma level of 50 µmol/L [4], which is the mean plasma level in UK adults [5]. This is sufficient to prevent scurvy but may be inadequate when a person is under viral exposure and physiological stress. An expert panel in cooperation with the Swiss Society of Nutrition recommended that everyone supplement with 200 mg “to fill the nutrient gap for the general population and especially for the adults age 65 and older. This supplement is targeted to strengthen the immune system” [6]. The Linus Pauling Institute recommends 400 mg for older adults (>50 years old) [7].

Pharmacokinetic studies in healthy volunteers support a 200 mg daily dose to produce a plasma level of circa 70 to 90 µmol/L [8,9]. Complete plasma saturation occurs between 1 g daily and 3 g every four hours, being the highest tolerated oral dose, giving a predicted peak plasma concentration of circa 220 µmol/L [10]. The same dose given intravenously raises plasma vitamin C levels approximately ten-fold. Higher intakes of vitamin C are likely to be needed during viral infections with 2–3 g/day required to maintain normal plasma levels between 60 and 80 µmol/L [11,12]. Whether higher plasma levels have additional benefit is yet to be determined, but would be consistent with the results of the clinical trials discussed in this review.

## 2. Vitamin C Deficiency in Pneumonia, Sepsis and COVID-19

Human plasma vitamin C levels decline rapidly under conditions of physiological stress including infection, trauma, and surgery, not uncommonly resulting in overt vitamin C deficiency in hospitalised patients, defined as a plasma level of vitamin C ≤ 11 µmol/L [13,14,15,16,17,18]. Two studies in hospitals in Paris reported that 17 to 44% of patients had vitamin C plasma levels less than ≤ 11 µmol/L [14,15]. In a Canadian university hospital, it was found that 19% of patients had vitamin C plasma levels ≤ 11 µmol/L [16]. In a study of surgical patients in Australia, it was found that 21% had vitamin C plasma levels ≤ 11 µmol/L [17]. A survey of elderly Scottish patients hospitalised as a consequence of acute respiratory infections reported that 35% of patients had vitamin C plasma levels ≤ 11 µmol/L [18]. The UK’s National Diet and Nutrition Survey, based on a cross section of the UK population, reports that 4% of 65+ year olds and 40% of those institutionalised in care homes have vitamin C levels ≤ 11 µmol/L [5,19], indicating the way in which older people with low vitamin C status may be especially susceptible to critical infection.

The vitamin C-deficiency disease scurvy has long been associated with pneumonia which led to the view that vitamin C may influence susceptibility to respiratory infections [20]. In other words, people deficient in vitamin C may be more susceptible to severe respiratory infections such as pneumonia. A prospective study of 19,357 men and women followed over 20 years found that people in the top quartiles of baseline plasma vitamin C concentrations had a 30% lower risk of pneumonia [21]. Furthermore, meta-analysis has indicated a reduction in the risk of pneumonia with oral vitamin C supplementation, particularly in individuals with low dietary intakes [22].

Post-mortem investigations of severe COVID-19 have demonstrated a secondary organising pneumonia phenomenon [23]; therefore, studies investigating vitamin C in relation to pneumonia may be relevant [18,24,25,26,27] (Table 1). The most recent study, from New Zealand, reported that patients with pneumonia had depleted vitamin C levels compared with healthy controls (23 µmol/L vs. 56 µmol/L, *p* < 0.001). The pneumonia cohort comprised 62% with hypovitaminosis C and 22% with vitamin C ≤ 11 µmol/L, compared with 8% hypovitaminosis C and no cases with ≤11µmol/L in the healthy controls [24]. The more severely ill patients in the ICU had mean vitamin C levels of 11 µmol/L. Similar findings have been reported in other studies of critically ill septic patients [28,29,30,31,32,33] (Table 1). A New Zealand study of patients with sepsis found that 40% had vitamin C ≤ 11 µmol/L and the majority of the patients had hypovitaminosis C (serum level < 23 µmol/L), despite receiving recommended enteral and parenteral intakes of the vitamin [29].

As yet, there have been few studies reporting the vitamin C status of patients with COVID-19 (Table 1). A study of 21 critically ill COVID-19 patients admitted to ICU in the US found a mean level of 22 µmol/L, thus a majority had hypovitaminosis C. The mean level for 11 survivors was 29 µmol/L compared to 15 µmol/L for the 10 non-survivors; of these five (50%) had ≤11 µmol/L [34]. A study in an ICU in Barcelona of 18 COVID-19 patients meeting acute respiratory distress syndrome (ARDS) criteria found that 17 had undetectable levels of vitamin C (i.e., <9 µmol/L) and one patient had a low vitamin C (14 µmol/L) [35]. Thus, low vitamin C levels are common in critically ill hospitalised patients with respiratory infections, pneumonia, sepsis and COVID-19, the most likely explanation being increased metabolic consumption [37].

## 3. Mechanisms of Action of Vitamin C in Infections, Sepsis and COVID-19

Vitamin C has important anti-inflammatory, immunomodulating, antioxidant, antithrombotic and antiviral properties [38,39,40]. The vitamin demonstrates direct virucidal activity and has effector mechanisms in both the innate and adaptive immune systems [41,42,43,44]. The effects of vitamin C on immunity during infection are many and include the development and maturation of T-lymphocytes and the functions of phagocytosis and chemotaxis of leucocytes [45]. It also has a vital role as an antioxidant whereby phagocytes import oxidised vitamin C (dehydroascorbic acid) and regenerate it to reduced vitamin C (ascorbic acid) [46,47]. 

Importantly, and with specific reference to the critical phase of COVID-19, vitamin C contributes to the downregulation of cytokines, protecting the endothelium from oxidant injury and has an essential role in tissue repair [48,49]. The interaction between oxidative stress and the induction of genes integral to the inflammatory response, including TNFα, IL-1, IL-8, and ICAM-1 has been shown to be mediated through activation of NF-κB [50]. Vitamin C lessens reactive oxidative species (ROS) and inflammation via attenuation of NF-κB activation [51]. Vitamin C significantly increases superoxide dismutase, catalase and glutathione and decreases serum TNFα and IL-1β levels in a rat ARDS model [52]. These effects of vitamin C may be due to its epigenetic regulation of various genes, i.e., upregulation of antioxidant proteins and downregulation of proinflammatory cytokines, rather than its direct scavenging of oxidants. 

While SARS-CoV-2 downregulates the expression of type-1 interferons (the host’s primary anti-viral defence mechanism) [53], vitamin C upregulates these key host defence proteins [40]. In GULO knockout mice, vitamin C shows in vivo anti-viral immune responses and a reduction in viral titres in the lung in the early stage of infection, especially against influenza virus, through increased production of interferon [54]. Animal studies show that vitamin C reduces the incidence and severity of bacterial and viral infections [55], including increased resistance of chick embryo tracheal organ cultures to coronavirus infection and protection of broiler chicks against avian coronavirus [56,57].

Based on the identification of ACE2 as the receptor for SARS-CoV-2 entry, there is a hypothesis that the increased risk of severe COVID-19 is a function of upregulated ACE2, as is found in the co-morbidities of diabetes, cardiovascular disease and hypertension [58]. The SARS-CoV-2 spike glycoprotein is able to bind to ACE2 [59]. It is noteworthy that, in human arterial endothelial cells, vitamin C abolished ACE2 upregulation induced by IL-7 [60].

Although there are many potential targets for vitamin C in the process of infection, viral replication and pathology in COVID-19, it is noteworthy that a key protease in the virus, Mpro, whose function is to activate several viral non-structural proteins, has been proposed as a target. In a modelling study using the crystal structure of Mpro, the active site of this enzyme was found to bind magnesium ascorbate, which had the strongest binding out of 106 nutraceuticals. The authors suggested that ascorbate might, therefore, be a powerful inhibitor of the enzyme [61].

The critical and often fatal phase of COVID-19, primarily triggered by the host’s reaction to dead virus particles, occurs with increased production of potent proinflammatory cytokines and chemokines, resulting in the development of multi-organ failure [62]. This may result in neutrophil migration and accumulation in the lung interstitium and bronchoalveolar space and is considered a key determinant of progression of ARDS [63]. Neutrophil extracellular trap formation (NETosis) is a cell death pathway different from apoptosis and necrosis that traps and inactivates pathogens [64]. This is a maladaptive response that may contribute to tissue and organ damage leading to organ failure. Vitamin C deficiency in GULO-knockout mice showed enhanced NETosis in the lungs of septic animals and increased circulating cell-free DNA suggesting that vitamin C is a novel regulator of NETosis [65]. Furthermore, vitamin C enhances lung epithelial barrier function in an animal model of sepsis by promoting epigenetic and transcriptional expression of protein-channels at the alveolar capillary membrane that regulate alveolar fluid clearance which include cystic fibrosis transmembrane conductance regulator, aquaporin-5, the Na^+^/K^+^-ATPase pump and epithelial sodium channel [66].

**Figure 1:**
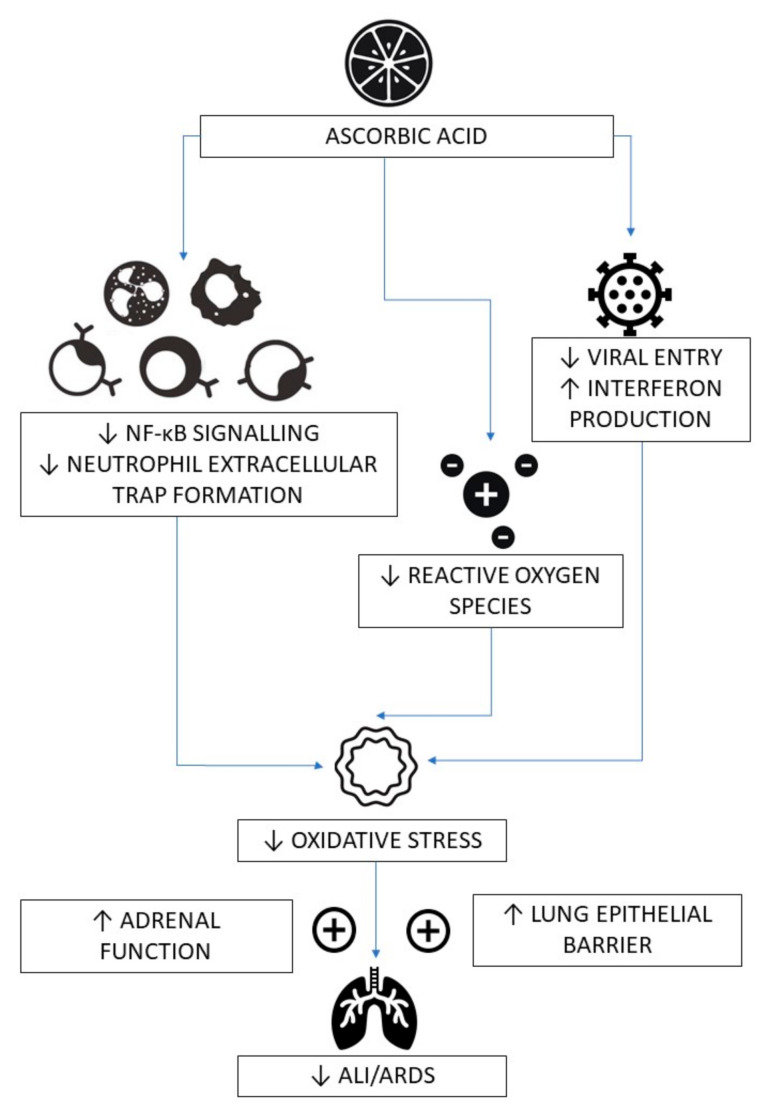
Postulated mechanisms for vitamin C’s amelioration of COVID-19 pathology. ↓—decreased; ↑—increased; ALI—acute lung injury; ARDS—acute respiratory distress syndrome; NF-κB—nuclear factor kappa B.

There is also increasing evidence that vitamin C, which is a pleiotropic stress hormone, may be playing a critical role in mediating the adrenocortical stress response, particularly in sepsis [38]. Vitamin C concentrations are three to ten times higher in the adrenal glands than in any other organ [67]. It is released from the adrenal cortex under conditions of physiological stress (ACTH stimulation), including viral exposure, raising plasma levels fivefold [68]. Vitamin C enhances cortisol production and potentiates the anti-inflammatory and endothelial cytoprotective effects of glucocorticoids [69,70]. Exogenous glucocorticoid steroids are the only proven disease-modifying treatment for COVID-19 [71]. The postulated mechanisms for vitamin C’s amelioration of COVID-19 pathology are shown in Figure 1.

## 4. Clinical Evidence for the Role of Vitamin C in Colds

Nobel laureate Linus Pauling concluded from randomised controlled trials (RCTs) that vitamin C prevented and alleviated colds thus popularising its use in the 1970s [72,73]. A Cochrane Review of placebo-controlled trials giving oral vitamin C for preventing and treating colds found that supplementation above 200 mg did not reduce the incidence in the general population [74]. However, in five trials involving a total of 598 marathon runners, skiers and soldiers on subarctic exercises vitamin C reduced the incidence of colds by 52% (*p* < 0.0001) [74]. Based on these findings, vitamin C appears to influence resistance to viral infections in special conditions, such as during brief periods of severe physical exercise.

Whereas trials where vitamin C has been administered only after the onset of symptoms have not shown consistent benefits, trials which regularly administered vitamin C reduced the duration of infections in adults by 8% and in children by 14%, with an apparent dose-dependency up to 6–8 g/day [55,74]. In children, 1 to 2 g/day vitamin C reduced cold duration by 18%, with the severity of colds being reduced by regular administration [74].

The latest UK placebo-controlled trial illustrates the meaningful clinical difference between the number of colds, cold duration and severity [75]. This trial comprised 168 volunteers who were randomised to receive a placebo or vitamin C (2 × 500 mg daily) over a 60-day winter period. The vitamin C group had fewer colds (37 vs. 50, *p* = 0.05), and even fewer virally challenged ‘cold’ days (85 vs. 178, *p* = 0.03) and a shorter duration of severe symptom days (1.8 vs. 3.1 days, *p* = 0.03). The number of participants who had two colds during the trial was significantly reduced (2/84 on vitamin C vs. 16/84 in the placebo group; *p* = 0.04) [75]. 

In summary, cold symptoms have been shown to be less severe and resolve more quickly with oral vitamin C with a dose-dependent effect. Colds, caused by over 100 different virus strains, some of which are coronaviruses, are defined by a group of symptoms similar to the majority of those who get SARS-CoV-2 infection and do not convert into the acute illness phase. This similarity of symptoms and the disease-modifying effect of vitamin C across a wide range of cold-related viruses is further rationale for considering that vitamin C’s effects in reducing severity and duration of infection is not virus-specific and could thus also potentially alleviate SARS-CoV-2 related symptoms. Each of these effects—reduced duration, severity and number of colds—could reasonably be hypothesised, in the context of SARS-CoV-2, to reduce conversion from mild infection to the critical phase of COVID-19. Given the consistent effect of regular vitamin C intake on the duration and severity of colds, and the low cost and safety, it would be appropriate for patients with respiratory virus infections to have the benefits of therapeutic vitamin C assessed.

Since the disease caused by the novel coronavirus can be more severe than ordinary viral infections, the above estimates may justify a regular increased daily intake of vitamin C for the period when the prevalence of the virus is high, when a patient suffers from a virus infection with active cold symptoms, in those testing PCR positive to SARS-CoV-2 and in COVID-19 hospitalised patients; an oral dose of up to 6–8 g/day might be considered. Pauling’s recommendation of 1 g every hour of oral ascorbic acid during active infection has yet to be studied in an RCT, therefore, the most effective dose has yet to be determined.

## 5. Clinical Evidence for the Role of Vitamin C in Pneumonia

In 1951, Klenner investigated the effects of high doses of vitamin C, given intravenously, against viral diseases including pneumonia [76]. A Cochrane review on pneumonia and vitamin C identified three prophylactic RCTs reporting the number of pneumonia cases in participants who were administered oral vitamin C [22]. Each of these found a ≥80% lower incidence of pneumonia for the vitamin C group [77,78,79]. One was an RCT giving 2 g/day versus placebo to US Marine recruits during a two-month recruit training period and reported 1/331 cases of pneumonia in the vitamin C group versus 7/343 cases in the placebo group (*p* = 0.044) [77]. 

Two therapeutic trials were identified (Table 2). One was an RCT with elderly people in the UK (mean age 81 years), hospitalised with acute bronchitis or pneumonia. The study found that the plasma vitamin C level at baseline was 23 µmol/L (hypovitaminosis C) and one third of the patients had a vitamin C level of ≤11 µmol/L [18]. Vitamin C (0.2 g/day) reduced the respiratory symptom score in the more ill patients but not the less ill. There were six deaths during the study, all among the more ill patients: five in the placebo group, but only one in the vitamin C group. The other RCT, in the former Soviet Union, administered two different doses, a variable high or low dose relating to the dosage of antibiotics given [27]. The duration of hospital stay in the control group was 23.7 days. In the low dose vitamin C group (0.25–0.8 g/day) hospital stay was 19% shorter and in the high-dose group (0.5–1.6 g/day) it was 36% shorter. A benefit was also reported in relation to erythrocyte sedimentation rate and the normalisation of chest X-ray and temperature. 

## 6. Clinical Evidence for the Role of Vitamin C in Critically Ill Septic Patients

The major cause for concern regarding COVID-19 is the high frequency of ICU treatment that is needed. Meta-analyses of intravenous vitamin C supplementation in critically ill (burns, sepsis and septic shock) patients indicated that it can lead to vasopressor sparing effects, reduced duration of ICU stay and a reduced need for mechanical ventilation [83]. In six trials, orally administered vitamin C in doses of 1–3 g/day reduced the length of ICU stay by 8.6% (*p* = 0.003) [84]. In five trials including 471 patients requiring ventilation for over 10 h, a dosage of 1–6 g/day of vitamin C reduced ventilation time by 25% (*p* < 0.0001) [85].

There is clear evidence that vitamin C levels decline precipitously in critically ill patients and in those with sepsis (Table 1) [36]. Although 0.1 g/day of vitamin C can maintain a normal plasma level in a healthy person, much higher doses (2–3 g/day) are needed to keep plasma vitamin C levels of critically ill patients within the normal range [11,86]. Being water-soluble, and thus excreted within hours, frequency of dose is important to maintain sufficient blood levels during active infection. Limitations in bioavailability in conditions of rapid vitamin C depletion in critically unwell patients have generated the hypothesis that the required therapeutic plasma levels to optimally reduce oxidative stress and exert an anti-inflammatory effect are more effectively achieved with intravenous administration than with oral administration alone [29,87]. 

Clinicians using intravenous vitamin C in severely ill COVID-19 patients have reported clinical effects upon administration of 3 g every 6 h together with steroids and anti-coagulants [88]. However, clear evidence for the most effective dose and frequency has not yet been determined. A four-group randomised pharmacokinetic trial testing 2 or 10 g/day, either delivered as a twice-daily bolus infusion or continuous infusion, found that the 2 g/day dose was associated with normal plasma concentrations, and the 10 g/day dose was associated with supranormal plasma concentrations, increased oxalate excretion, and metabolic alkalosis. The study’s authors also concluded that sustained therapy is needed to prevent hypovitaminosis C [11]. 

Vitamin C has been reported to reduce mortality in septic patients requiring vasopressor treatment randomly assigned to be given 25 mg/kg body weight/day intravenous vitamin C every 6 h versus placebo (Table 2). Mortality at 28 days was significantly lower in the ascorbic acid than the placebo group (14% vs. 64%, respectively; *p* = 0.009) [81].

In the largest trial of intravenous vitamin C in sepsis-associated ARDS, the CITRIS-ALI trial, patients were given placebo or vitamin C at a dose of 50 mg/kg every 6 h for 4 days, thus providing 15 g/day for a 75 kg person (Table 2). Patients in the vitamin C group did not have significantly improved markers of inflammation, vascular injury or organ dysfunction which were the primary outcomes [28]. However, there were statistically significant benefits in three of the four clinically relevant outcomes, i.e., mortality (*p* = 0.03), duration of ICU-free days (*p* = 0.03) and hospital-free days (*p* = 0.04). Reanalysis of the data indicated that, during the 4-day vitamin C administration, mortality was 81% lower, but after the cessation of vitamin C administration, there was no difference between the two trial groups [89]. By the end of the 4-day vitamin C administration, the mortality rate was 23% (19/83) in the placebo group and 5% (4/84) in the vitamin C group (*p* = 0.0007). This difference of 18% corresponds to the number needed to treat of 5.5. Furthermore, the study authors, in recognition of the exclusion of sequential organ failure assessment (SOFA) scores in deceased patients, reported in a post hoc analysis assigning deceased patients a SOFA score of 20 and discharged patients a SOFA score of zero, that there was a 60% probability that any random patient from the placebo group had a higher SOFA score than any random patient from the vitamin C group (*p* = 0.03) at 96 h [90]. 

Another trial randomised 216 patients to low-dose intravenous vitamin C (1.5 g every 6 h thus providing 7.5 g/day), thiamine, and hydrocortisone for up to 10 days or until septic shock resolved, with a mean of 3.4 days, versus hydroxycortisone alone, and found no effect on the primary outcome of vasopressor-free time to 7 days or on 90-day mortality [91]. Two limitations of this study are the delay in giving vitamin C [92], and the absence of a vitamin C only arm [93]; hence, this study only shows that the addition of vitamin C, possibly too late in the disease process and for too short a time, to hydroxycortisone treatment added no treatment advantage.

## 7. Clinical Evidence for the Role of Vitamin C in COVID-19 

Given the potential benefit of vitamin C, in oral and intravenous doses of 2–8 g/day, to reduce duration and severity of the common cold, pneumonia, sepsis and ARDS, this warrants investigation in relation to whether early oral supplementation could be beneficial in preventing conversion from mild infection to more critical COVID-19 infection and, if given intravenously to those with critical COVID-19 symptoms, in reducing mortality and ICU stay, thus speeding up recovery. 

Interestingly, many of the risk factors for COVID-19 overlap with those for vitamin C deficiency [94]. Certain sub-groups (male, African American, older, those suffering with co-morbidities of diabetes, hypertension, COPD), all at higher risk of severe COVID-19, have also been shown to have lower serum vitamin C levels [95]. Average plasma vitamin C levels are generally lower in men than women, even with comparative intakes of vitamin C, which has been attributed to their higher body weight [94]. A hypothesis of altered sodium-dependent vitamin C transporter (SVCT1 and 2) expression in these sub-groups has also been proposed [95]. In old versus young rat hepatocytes, the vitamin C level declines by 66%, which is largely attributed to reduced absorption due to a 45% decline in SVCT1 with age [96]. It is noteworthy that inflammatory cytokines, also present in co-morbidities, downregulate SVCT2, resulting in the depletion of intracellular vitamin C [97,98].

**Figure 2:**
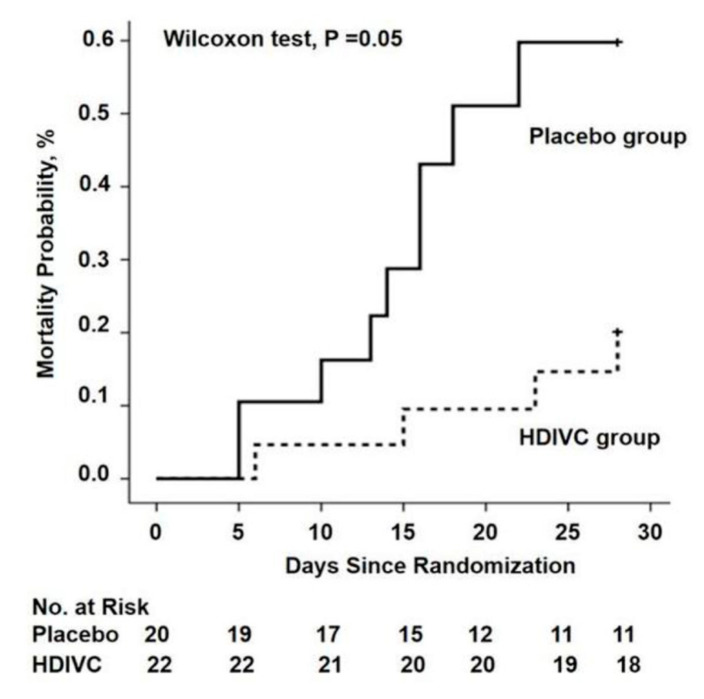
The 28-day mortality from randomization (day 1) to day 28 in a trial of high-dose intravenous vitamin C (HDIVC) in patients with COVID-19. Kaplan–Meier analysis was used to estimate the 28-day mortality and survival curves were compared with the Wilcoxon test (*p* = 0.05) among severe COVID-19 patients (baseline SOFA score ≥ 3). Cox regression was used as multiple comparisons (HR, 0.32 (95%CI, 0.10–1.06); *p* = 0.06). HDIVC—high-dose intravenous vitamin C. Reproduced with permission from Zhang J. et al. [82].

There are currently 45 trials registered on Clinicaltrials.gov investigating vitamin C with or without other treatments for COVID-19. In the first RCT to test the value of vitamin C in critically ill COVID-19 patients, 54 ventilated patients in Wuhan, China, were treated with a placebo (sterile water) or intravenous vitamin C at a dose of 24 g/day for 7 days [82] (Table 2). After 7 days of treatment, the ratio of PaO_2_/FiO_2_ in the vitamin C group was 229 mmHg versus 151 mmHg in the control group (*p* = 0.01), and this also improved over time in the vitamin C group, but fell in the control group. On day 7, the IL-6 level was lower in the vitamin C group than in the placebo group: 19 pg/mL versus 158 pg/mL (*p* = 0.04). The more severely ill patients with SOFA scores ≥ 3 in the vitamin C group exhibited a reduction in 28-day mortality: 18% versus 50% (*p* = 0.05) in univariate survival analysis (Figure 2). No study-related adverse events were reported. The effects of treatment on the ratio PaO_2_/FiO_2_ and on IL-6 are clinically important, but further studies are needed to determine if the trend in lower mortality can be confirmed. The trial was originally designed for 140 subjects and was thus underpowered, with only 54 patients due to a lack of new admissions. 

The largest registered trial is the Lessening Organ Dysfunction with Vitamin C-COVID (LOVIT-COVID) trial in Canada, which is recruiting 800 patients who are randomly assigned to vitamin C (intravenous, 50 mg/kg every 6 h) or a placebo for 96 h, i.e., equivalent to 15 g/day for a 75 kg person (NCT04401150). This protocol has also been added as a vitamin C arm in the Randomized, Embedded, Multifactorial Adaptive Platform Trial for Community-Acquired Pneumonia (REMAP-CAP; NCT02735707). The study design provides further rationale for the use of vitamin C in COVID-19 patients [99]. There is also a high-dose (10 g/day) vitamin C intervention study in 500 adults is in progress in Palermo, Italy (NCT04323514).

There is concern, however, that these study designs limit the use of vitamin C to a maximum of four days, which may be inadvisable in acutely ill patients due to the potential return of symptoms if the inflammation is not resolved. This issue was illustrated by the CITRIS-ALI trial, which showed a maximum reduction in mortality compared to placebo on day 4, the final day of vitamin C administration, but a decreased difference between the groups after 28 days [87,89].

In the UK, the Chelsea and Westminster hospital ICU, where adult ICU patients were administered 1 g of intravenous vitamin C every 12 h together with anticoagulants [100], has reported 29% mortality [101], compared to the average 41% reported by the Intensive Care National Audit and Research Centre (ICNARC) for all UK ICUs [102]. While the authors have stated that the addition of an antioxidant in the form of vitamin C could have contributed to the lower mortality rate, it should be noted that other clinical factors and procedures could also account for the improved mortality and that the Chelsea and Westminster ICU serves a more affluent sector of the population with less deprivation on the basis of the Index of Multiple Deprivation (IMD). Deprivation, while a risk factor for COVID-19 mortality, is also a predictor of low vitamin C status. In the UK, an estimated 25% of men and 16% of women in the low-income/materially deprived population are deficient in vitamin C > 11 µmol/L [103].

The Frontline COVID-19 Critical Care Expert Group (FLCCC), a group of emergency medicine experts, have reported that, with the combined use of 6 g/day intravenous vitamin C (1.5 g every 6 h), plus steroids and anticoagulants, mortality was 5% in two ICUs in the US (United Memorial Hospital in Houston, Texas, and Norfolk General Hospital in Norfolk, Virginia), the lowest mortality rates in their respective counties [88].

A case report of 17 COVID-19 patients who were given 1 g of intravenous vitamin C every 8 h for 3 days reported a mortality rate of 12% with 18% rates of intubation and mechanical ventilation and a significant decrease in inflammatory markers, including ferritin and D-dimer, and a trend towards decreasing FiO_2_ requirements [104]. Another case of unexpected recovery following high-dose intravenous vitamin C has also been reported [105]. While these case reports are subject to confounding and are not prima facie evidence of effects, they do illustrate the feasibility of using vitamin C for COVID-19 with no adverse effects reported. 

## 8. Safety of Oral and Intravenous Vitamin C

The US DRI, having thoroughly considered the wide literature on vitamin C and many kinds of speculated harms, stated that the safe range is up to 2 g/day [106]. The European Food Safety Authority stated that the lowest observable adverse effect level is 3–4 g/day (in relation to gastrointestinal effects) [107]. Injectable vitamin C phials state “there are no contraindications to the administration of ascorbic acid. As much as 6 g has been administered parenterally to normal adults without evidence of toxicity” [108]. 

Three concerns have been raised regarding high doses of vitamin C: diarrhoea from high oral ingestion, kidney stones, particularly due to kidney dysfunction in the case of intravenous vitamin C (i.e., if high doses cannot be cleared), and unsuitability for those with specific genetically inherited metabolic issues that affect vitamin C utilisation. The latter relates to those with glucose-6-phosphate deficiency (G6PD) and also haemochromatosis and thalassaemia due to enhanced iron absorption with vitamin C. G6PD deficiency is not considered an exclusion criterion in the use of up to 6 g/day oral or intravenous vitamin C [109]. The FLCCC report that 3 g every 6 h appears to be safe in patients with G6PD. It may be wise for those with haemochromatosis or thalassaemia to avoid high-dose vitamin C taken with iron-rich foods or supplements and short-term high-dose vitamin C to be medically monitored [110].

Looser bowel movements and diarrhoea rarely occur below 3 g/day and tolerance is increased considerably when fighting a viral infection [111]. Diarrhoea has not been reported as a complication in hospital-based oral treatment and does not occur with intravenous vitamin C administration. A survey of 9328 patients given an average intravenous dose of 24 g of vitamin C every 4 days, primarily for cancer, infection or fatigue, reported that 101 (1%) had side effects, mostly minor, including lethargy/fatigue, a change in mental status and vein irritation/phlebitis [112].

Regarding kidney stone formation, the Kidney Stone Research Laboratory of the University of Cape Town conducted a controlled trial in which ten volunteer subjects were required to ingest 4 g of vitamin C per day for five days. Unlike the earlier studies, they put a preservative in the urine collection bottles to prevent the conversion of ascorbate to oxalic acid. The samples were analysed for numerous physicochemical risk factors of kidney stone formation. These risk factors were not significantly altered and the authors concluded that ingestion of large doses of vitamin C does not increase the risk of forming kidney stones and earlier trials had faulty study designs involving unpreserved urine samples [113]. A prospective cohort study of 85,557 women with no history of kidney stones, with 1078 incidences of kidney stones over 14 years of follow-up, reported that vitamin C was not associated with a risk of kidney stone development [114]. A systematic review of studies giving vitamin C found a correlation between ascorbic acid supplementation and the incidence of kidney stones in men, but not women [115]. A study administering intravenous ascorbic acid in doses ranging from 0.2 to 1.5 g/kg body weight measured urinary oxalic excretion during and over 6 h post infusion. The authors conclude that less than 0.5% of a very large intravenous dose of ascorbic acid was recovered as urinary oxalic acid in people with normal renal function [116]. A cautious position would be to exclude those with a history of kidney stones or kidney dysfunction from high-dose oral or intravenous vitamin C unless medically supervised. Short-term high-dose vitamin C in the region of 2–8 g/day is unlikely to be of significant concern in people with normal kidney function.

## 9. Conclusions

Vitamin C’s potential benefits, low cost, safety profile and multiple disease-modifying actions, including antioxidant, anti-inflammatory and immunomodulating effects, make it an attractive therapeutic candidate in reducing viral load with oral supplementation in the range of 2–8 g/day to help attenuate the conversion to the critical phase of COVID-19. Likewise, vitamin C has potential benefits in treating acute respiratory infections and mitigating inflammation in critical COVID-19 patients with intravenous vitamin C infusion in the range of 6–24 g/day, for correcting disease-induced deficiency, reducing inflammation, enhancing interferon production and supporting the anti-inflammatory actions of glucocorticosteroids, especially given the high level of fatality for patients with severe COVID-19.

Given the remarkable safety of vitamin C, frequent deficiency among patients with COVID-19 and extensive evidence of potential benefits, the current treatment is justified on compassionate grounds pending more COVID-19 clinical trial data becoming available, not only for intravenous use within ICUs, but also orally with doses between 2 and 8 g/day in hospitalised patients due to increased need when fighting a viral infection, as concluded in recent reviews [36,117,118]. The clinical choice of oral versus intravenous vitamin C may be guided by similar criteria for administering oral versus intravenous antibiotics, considering both the severity of the illness and whether the patient is able to swallow oral medication at least four times a day.

People in high-risk groups for COVID-19 mortality, and at risk of vitamin C deficiency, should be encouraged to supplement with vitamin C daily to ensure vitamin C adequacy at all times, and to increase the dose when virally infected to up to 6–8 g/day [119]. Whether or not this will prevent conversion to the critical phase of COVID-19 has yet to be determined.

## Figures and Tables

**Table 1:** Vitamin C status of patients with pneumonia, sepsis and severe COVID-19.

Study Type	Cohort	Vitamin C (µmol/L) (% Deficient, % Hypovitaminosis C)	Refs.
**Pneumonia**			
Case control	Healthy volunteers (*n* = 50)	56 ± 2 ^a^ (0% ^b^, 8% ^c^)	[24]
	Community-acquired pneumonia (*n* = 50)	23 ± 3 (22%, 62%)	
Case control	Healthy volunteers (*n* = 20)	66 ± 3	[25]
	Pneumonia cases (*n* = 11)	31 ± 9	
Case control	Healthy participants (*n* = 28)	49 ± 1	[26]
	Lobular pneumonia (*n* = 35):		
	Acute—did not survive (*n* = 7)	17 ± 1	
	Acute—survived (*n* = 15)	24 ± 1	
	Convalescent cases (*n* = 13)	34 ± 1	
Intervention (placebo group)	Pneumonia/bronchitis (*n* = 29):		[18]
	Week 0	24 ± 5 (40%) ^b^	
	Week 2	19 ± 3 (37%)	
	Week 4	24 ± 6 (25%)	
Intervention (control group)	Pneumonia cases (*n* = 70):		[27]
	Day 0	41	
	Day 5–10	23–24	
	Day 15–20	32–35	
	Day 30	39	
**Sepsis**			
Intervention (baseline)	Sepsis with ARDS (*n* = 83):		[28]
	Day 0	22 (11–37) ^d^	
	Day 2	23 (9–37)	
	Day 4	26 (9–41)	
	Day 7	29 (12–39)	
Observational	Septic shock patients (*n* = 24)	15 ± 2 (38% ^b^, 88% ^c^)	[29]
Intervention (baseline)	Severe sepsis patients (*n* = 24)	18 ± 2	[30]
Case control	Healthy controls (*n* = 6)	48 ± 6	[31]
	Severe sepsis (*n* = 19)	14 ± 3	
	Septic shock (*n* = 37)	14 ± 3	
Case control	Healthy controls (*n* = 14)	76 ± 6	[32]
	Septic encephalopathy (*n* = 11)	19 ± 11	
Case control	Healthy controls (*n* = 34)	62 (55–72) ^d^	[33]
	ICU (injury, surgery, sepsis) (*n* = 62)	11 (8–22)	
**Severe COVID-19**			
Observational	Critically ill COVID-19 (*n* = 21)	22 ± 4 (45%^b^, 70% ^c^) ^e^	[34]
	Survivors (*n* = 11)	29 ± 7 (40%, 50%)	
	Non-survivors (*n* = 10)	15 ± 2 (50%, 90%)	
Observational	COVID-associated ARDS (*n* = 18)	17 with <9 µmol/L	[35]
		1 with 14 µmol/L	

^a^—Data represent mean and SEM; ^d^—median (and interquartile range); ^b^—Percentage of patients with vitamin C deficiency (<11 µmol/L); ^c^—Percentage of patients with hypovitaminosis C (<23 µmol/L); ^e^—Personal communication (Cristian Arvinte, North Suburban Medical Center, Thornton, CO, USA). COVID—coronavirus disease; ICU—intensive care unit; ARDS—acute respiratory distress syndrome. A part of this table has been reproduced from [36].

**Table 2:** Vitamin C trials in patients with pneumonia, sepsis and severe COVID-19.

Patients	Intervention Dose (Duration)	Patient Outcomes	Refs.
**Pneumonia**			
Pneumonia/bronchitis (*n* = 57):	Oral vitamin C (28 day):	↓ respiratory symptom score in most severely ill	[18]
• Placebo (*n* = 29)	0 g/day	17% mortality in placebo group	
• Treatment (*n* = 28)	0.2 g/day	4% mortality in treatment group	
Pneumonia (*n* = 140):	Oral vitamin C (10 day):	↓ hospital length of stay:	[27]
• Control (*n* = 70)	0 g/day	24 days in control group	
• Low dose (*n* = 39)	0.25–0.8 g/day	19 days in low dose group	
• High dose (*n* = 31)	0.5–1.6 g/day	15 days in high dose group	
**Sepsis**			
Sepsis and ARDS (*n* = 167):	IV vitamin C (4 day):	X systemic organ failure score X C-reactive protein, thrombomodulin X ventilator-free days ↓ 28 day mortality ↑ ICU-free days ↑ hospital-free days	[28]
• Placebo (*n* = 83)	0 mg/kg bw/day		
• Treatment (*n* = 84)	200 mg/kg/day		
Septic shock (*n* = 100):	IV vitamin C (until ICU discharge)	↓ vasopressor duration ↓ ICU length of stay X length of mechanical ventilation X renal replacement therapy X ICU mortality	[80]
• Placebo (*n* = 50)	0 g/day		
• Treatment (*n* = 50)	6 g/day		
Septic shock (*n* = 28):	IV vitamin C (3 day):	↓ norepinephrine dose and duration ↓ 28 day mortality X ICU length of stay	[81]
• Placebo (*n* = 14)	0 mg/kg bw/day		
• Treatment (*n* = 14)	100 mg/kg bw/day		
Severe sepsis (*n* = 24)	IV vitamin C (4 day):	↓ systemic organ failure score ↓ C-reactive protein, procalcitonin, thrombomodulin	[30]
• Placebo (*n* = 8)	0 mg/kg bw/day		
• Low dose (*n* = 8)	50 mg/kg bw/day		
• High dose (*n* = 8)	200 mg/kg bw/day		
**Severe COVID-19**			
Critical COVID-19 (*n* = 54)	IV vitamin C (7 day):	X ventilation-free days ↑ PaO_2_/FiO_2_ ↓ Interleukin-6 ↓ 28 day mortality in patients with SOFA scores ≥ 3	[82]
• Placebo (*n* = 28)	0 g/day		
• Treatment (*n* = 26)	24 g/day		

ARDS—acute respiratory distress syndrome; COVID—coronavirus disease; FiO_2_—fraction of inspired oxygen; IV—intravenous; PaO_2_—partial pressure of oxygen; SOFA—sequential organ failure assessment; ↓—decrease; X—no change. A part of this table has been reproduced from [36].
